# *ω*-3 PUFAs and Resveratrol Differently Modulate Acute and Chronic Inflammatory Processes

**DOI:** 10.1155/2015/535189

**Published:** 2015-08-02

**Authors:** Joseph Schwager, Nathalie Richard, Christoph Riegger, Norman Salem

**Affiliations:** ^1^DSM Nutritional Products Ltd., Department of Human Nutrition & Health, P.O. Box 2676, 4002 Basel, Switzerland; ^2^DSM Nutritional Products Ltd., 6480 Dobbin Road, Columbia, MD 21045, USA

## Abstract

*ω*-3 PUFAs and polyphenols have multiple effects on inflammation *in vivo* and *in vitro*. The effects of eicosapentaenoic acid (EPA), docosahexaenoic acid (DHA), and resveratrol (RV) were investigated in LPS-stimulated peripheral blood leukocytes (PBLs) (i.e., acute inflammation) and IL-1*β* activated human chondrocytes (i.e., chronic inflammation). Inflammatory mediators including chemokines, cytokines, interleukins, and PGE_2_ were measured by multiplex analysis and gene expression was quantified by RT-PCR. In PBLs, RV decreased the secretion of PGE_2_, CCL5/RANTES, and CXCL8/IL-8 but increased IL-1*β*, IL-6, and IL-10. In contrast to RV, *ω*-3 PUFAs augmented the production of PGE_2_ and CXCL8/IL-8. EPA and DHA similarly affected the pattern of inflammatory mediators. Combination of RV and *ω*-3 PUFAs exerted synergistic effects on CCL5/RANTES and had additive effects on IL-6 or CXCL8/IL-8. Both *ω*-3 PUFAs and RV reduced catabolic gene expression (e.g., MMPs, ADAMTS-4, IL-1*β*, and IL-6) in activated chondrocytes. The data suggest that *ω*-3 PUFAs and RV differ in the regulation of acute inflammation of peripheral blood leukocytes but have common properties in modulating features related to chronic inflammation of chondrocytes.

## 1. Introduction

Cells and tissues respond to changes in physiological milieu as well as to external insults. Acute inflammatory processes are part of the normal response of the organism and are indispensable to restoring homeostasis. This is achieved by multiple mediators that initiate and modulate the extent and duration of and also resolve inflammatory processes. When these feedback mechanisms fail or are dysregulated, inflammatory mediators might remain in a status of low-grade or chronic inflammation as observed in obesity, cardiovascular diseases (CVD), diabetes, and arthritis. Cells of the immune system including peripheral blood leukocytes (PBLs) have a key role in the regulation of acute and chronic inflammation. They are continuously exposed to various nutrients, which can therefore influence their metabolic and functional status. Food components like *ω*-3 polyunsaturated acids (PUFAs) or micronutrients such as vitamins and antioxidants were found to beneficially modulate inflammatory processes and diseases [1–3]. Specifically, eicosapentaenoic acid (EPA) and docosahexaenoic acid (DHA) modulate eicosanoid metabolism and are precursors of mediators that are essential for the resolution of inflammation [4]. The anti-inflammatory effects of *ω*-3 PUFAs have been demonstrated in cellular systems and human studies [5–16] (see also [17] for review). On the other hand,* in vitro* activated chondrocytes are an adequate cellular model to analyse potential effects of nutrients on cells such as chondrocytes at conditions of chronic inflammation.

In this study we investigated the effects of *ω*-3 PUFAs on a panel of inflammatory mediators (cytokines, interleukins, and chemokines) produced by activated PBL from healthy individuals and by stimulated human chondrocytes and compared them to the natural phenolic compound resveratrol (RV) that has multiple effects on the inflammatory response [18]. We show that the substances have distinct effects on leukocytes and chondrocytes, which reflect acute and chronic inflammatory processes, respectively.

## 2. Materials and Methods

### 2.1. PUFAs, Resveratrol, and Cell Culture Reagents

Free fatty acid eicosapentaenoic acid (EPA), docosahexaenoic acid (DHA), and resveratrol were from Sigma/Aldrich (Saint Louis, MO).* E. coli* lipopolysaccharide (LPS, serotype 055:B5) and fetal bovine serum (FBS) were from Sigma/Aldrich (Saint Louis, MO). Cell culture reagents (RPMI 1640, 2-mercaptoethanol, and MEM nonessential amino acids (NEAA)) were from Invitrogen (Carlsbad, CA). IL-1*β* was purchased from PeproTech EC (London, UK). Substances were dissolved in DMSO and added to cells shortly before treating the cells with biological stimulators. Final concentrations of PUFAs and resveratrol were 10–50 *μ*mol/L and 1.25–50 *μ*mol/L, respectively.

### 2.2. Cell Culture

Human peripheral blood leukocytes (PBLs) were isolated from buffy coats obtained from healthy individuals and treated with inflammatory stimuli as described [19]. Briefly, PBLs were cultured in phenol red-free RPMI 1640 (containing 0.25% FBS, 0.1 mM NEAA, 50 U/mL penicillin, and 50 *μ*mg/mL streptomycin) and stimulated with 1 *μ*g/mL LPS in the presence of graded amounts of substances. Human* in vitro* PBL experiments were approved by the Swiss Federal Office of Public Health (number A050573/2) and the Ethical Commission of the Kanton Aargau, Switzerland. Cells were lysed in RLT buffer (Qiagen, Hilden, Germany) after 12 h of culture and total RNA was extracted. Alternatively, cells were cultured for 24 h; and secreted mediators were analyzed in culture supernatants.

Normal human articular chondrocytes from knee (NHAC-kn), prepared from different individuals, were cultured in chondrocyte growth medium (Lonza, Walkersville, MD) and used for experiments between passages 3 and 6 [20]. Cells (0.5 × 10^6^ per well) were seeded into 6-well plates and confluent cells were activated with IL-1*β* (10 ng/mL) in the presence of graded concentrations of test substances for 4–24 h [20].

### 2.3. RNA Isolation, cDNA Synthesis, and RT-PCR

The isolation of total RNA, synthesis of cDNA, and quantitative RT-PCR have been detailed previously [19, 20].

### 2.4. Multiparametric Analysis of Cytokines, Chemokines, and Interleukins

Multiparametric kits were purchased from Bio-Rad Laboratories (Hercules, CA) and used in the LiquiChip Workstation IS 200 (Qiagen, Hilden, Germany) to measure the amount of secreted proteins. Data evaluation was done using the LiquiChip Analyser software (Qiagen). PGE_2_ were measured as described previously [20].

### 2.5. Statistical Analysis

Data were evaluated by statistical tools described previously [20]. A *p* value <0.05 (calculated by using Student's *t*-test or one-way ANOVA) was considered to reflect statistically significant differences.

### 2.6. Calculation of Combination Effects

The algorithm developed by Chou and Talalay has been used to calculate synergism of inhibitory effects [21, 22]. Interactions were quantified by the combination index (CI) as described by Pappa et al. [23]: Using CalcuSyn software (Biosoft, Ferguson, MO), a CI was computed for every fraction affected. CI < 1 reflects synergistic inhibition of the respective inflammatory parameter; if CI = 1 the substances have additive interactions; when CI > 1 the interaction of substances reflects antagonism.

## 3. Results

### 3.1. Resveratrol and *ω*-3 PUFAs Modulate the Production of Cytokines and Chemokines in Leukocytes

**Figure 1:**
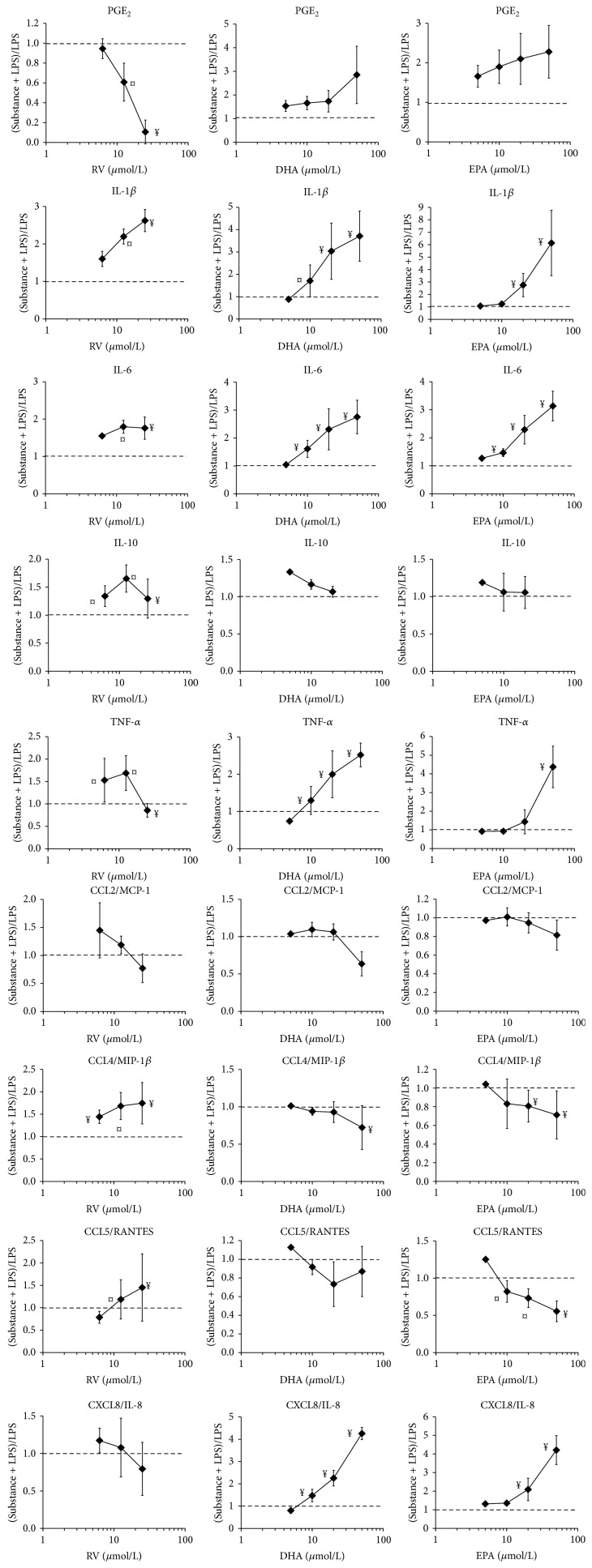
Production of inflammatory mediators by activated PBLs and its modulation by RV, DHA, and EPA. PBLs from healthy individuals were activated with LPS in the presence of the indicated substances (in *μ*mol/L) and the inflammatory mediators in supernatants of 24 h cultures were determined in duplicate. The results are expressed as the ratio of substance + LPS-treated cells/LPS-treated cells. Data are given as mean ± SD of experiments obtained from PBL from at least four individuals. The dotted line (at ratio 1) indicates the no-effect level. ¤, ¥ indicate statistically significant differences observed in 50% and >75% of the donors, respectively.

In order to investigate the effects of nutrients on acute inflammatory responses, PBLs from different individuals were activated with LPS in the presence of graded amounts of substances. LPS triggered substantial secretion of cytokines, chemokines, and PGE_2_; some of these differed considerably between individuals with respect to IL-1*β*, TNF-*α*, and CCL5/RANTES showing the largest interindividual variations (Table 1). The effects of resveratrol (RV) and *ω*-3 PUFAs on the inflammatory mediators of activated PBLs are shown in Figure 1. In order to correct for interindividual variations, the data are expressed as a ratio [(substance + LPS-treated cells)/LPS-treated cells]. RV drastically reduced the secretion of PGE_2_, which is dependent on the LPS-induced expression of COX-2 in monocytes/macrophages (Table 1). Conversely, interleukin- (IL-) 1*β*, IL-6, and the anti-inflammatory IL-10 were increased in the presence of RV (6.25–25 *μ*mol/L). We further investigated the impact of RV on chemokine secretion. CCL5/RANTES, which recruits activated T lymphocytes [24], was augmented by high concentrations of RV (25 *μ*mol/L). The neutrophil recruiting chemokine CXCL8/IL-8 was blunted by increasing RV concentration. CCL2/MCP-1, which is involved in targeted migration of resident monocytes [24] and macrophage polarization [25], was not significantly altered. In contrast, RV enhanced production of CCL4/MIP-1*β*, a chemokine for subtypes of monocytes [26].

DHA modulated the production of inflammatory mediators of LPS-activated leukocytes in a different manner (Figure 1). It enhanced PGE_2_ production (at 5–20 *μ*mol/L). IL-1*β* and IL-6 were produced to larger extents when DHA was included in the cellular assay. Similarly, DHA markedly enhanced CXCL8/IL-8 secretion, whereas it mitigated CCL5/RANTES. The production of CCL2/MCP-1 and CCL4/MIP-1*β* by activated leukocytes was only influenced by high concentrations of DHA. EPA shared with DHA a similar activity pattern on the production of inflammatory mediators. It should be noted that the extent of the response of PBLs to LPS was subject to large interindividual variations that presumably mirrored the differing immune status of the donors (Table 1).

### 3.2. Altered Gene Expression in Activated PBLs

**Figure 2:**
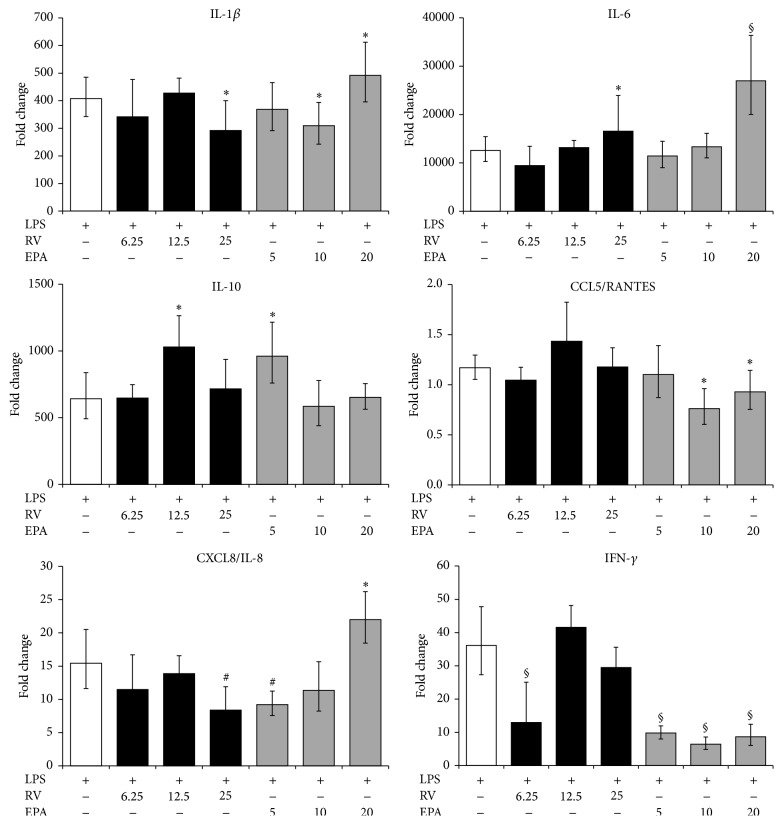
Expression of inflammatory genes in activated PBLs. Cells were activated with LPS with or without the indicated substances for 12 h. Gene expression was quantified by RT-PCR and the data expressed as fold change compared to levels observed in unstimulated cells. Mean ± standard errors of duplicates from three individuals are given. ^∗^ *p* < 0.05, ^#^ *p* < 0.01, and ^§^ *p* < 0.001 (LPS only versus LPS + substance).

By using quantitative RT-PCR, we investigated the impact of the substances on the transcription of inflammatory genes in PBLs after 12 h of LPS-stimulation, when many LPS-responsive genes were still upregulated [19, 27]. RV had only a minor influence on IL-1*β* mRNA levels, whereas it significantly augmented IL-6 transcription (Figure 2), consistent with the increased IL-6 secretion (Figure 1). Similarly, IL-10 mRNA levels of RV-treated cells matched the higher secretion of IL-10. We observed a significant decrease of CXCL8/IL-8 expression by RV (at 25 *μ*mol/L) (Figure 2). Conversely, CCL5/RANTES gene expression was not induced by LPS-stimulation nor changed by concomitant RV treatment. DHA altered gene expression patterns in a similar way, as it changed the secretion of the respective proteins: IL-6 expression and, to a lesser extent, IL-1*β* and IL-10 expression were augmented by DHA (at 20 *μ*mol/L) (Figure 2). CXCL8/IL-8 expression was enhanced, when DHA was included at 20 *μ*mol/L in the assay. Collectively, the data indicate that RV and *ω*-3 PUFAs regulate cytokine and chemokine production at the level of transcription.

### 3.3. Effects of Combinations of *ω*-3 PUFAs and RV Measured in PBLs

**Figure 3:**
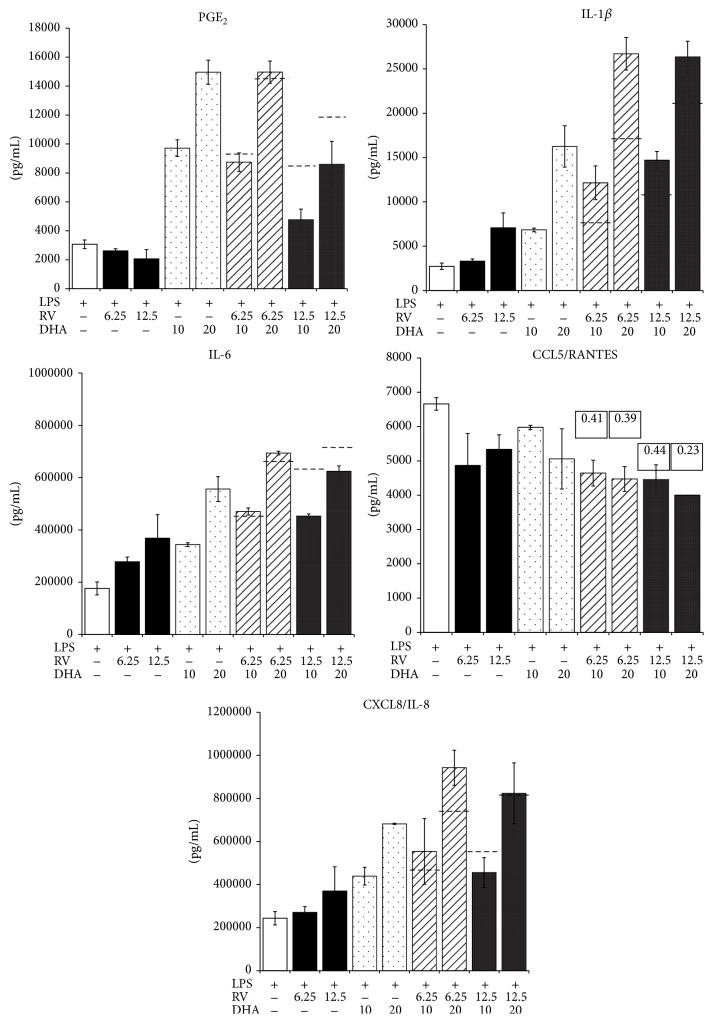
Effect of combinations of substances on inflammatory mediators produced by activated PBLs. PBLs were cultured for 24 h with or without the indicated substances and their combinations. In the case of* inhibition*, the combination index (CI) was calculated and is indicated in the figure. Dashed lines in the bar graphs indicate the computed sum of the respective single substance treatments.

Since we observed similar and opposite effects of substances, we investigated the pattern on inflammatory parameters produced when PBLs were treated with a combination of substances. To this aim, cells were activated in the presence of different concentrations and ratios of individual substances and the secreted mediators determined (Figure 3). PGE_2_ production was dominated by the inhibitory effect of RV, which partially counterbalanced the enhancing effect of DHA (Figure 3). CXCL8/IL-8 production was controlled by DHA, since the combined treatment with RV did not result in an intermediate production. Combinations of RV and DHA synergistically inhibited CCL5/RANTES secretion, as computed by the Chou-Talalay algorithm (Figure 3). Both RV and DHA concentration-dependently enhanced IL-6 secretion and combinations thereof had additive effects. IL-1*β*, however, appeared to be synergistically enhanced by RV and DHA, since the effect of combined substances largely exceeded the sum of RV and DHA applied individually.

### 3.4. Effects of *ω*-3 PUFAs and RV on IL-1*β* Activated Chondrocytes

**Figure 4:**
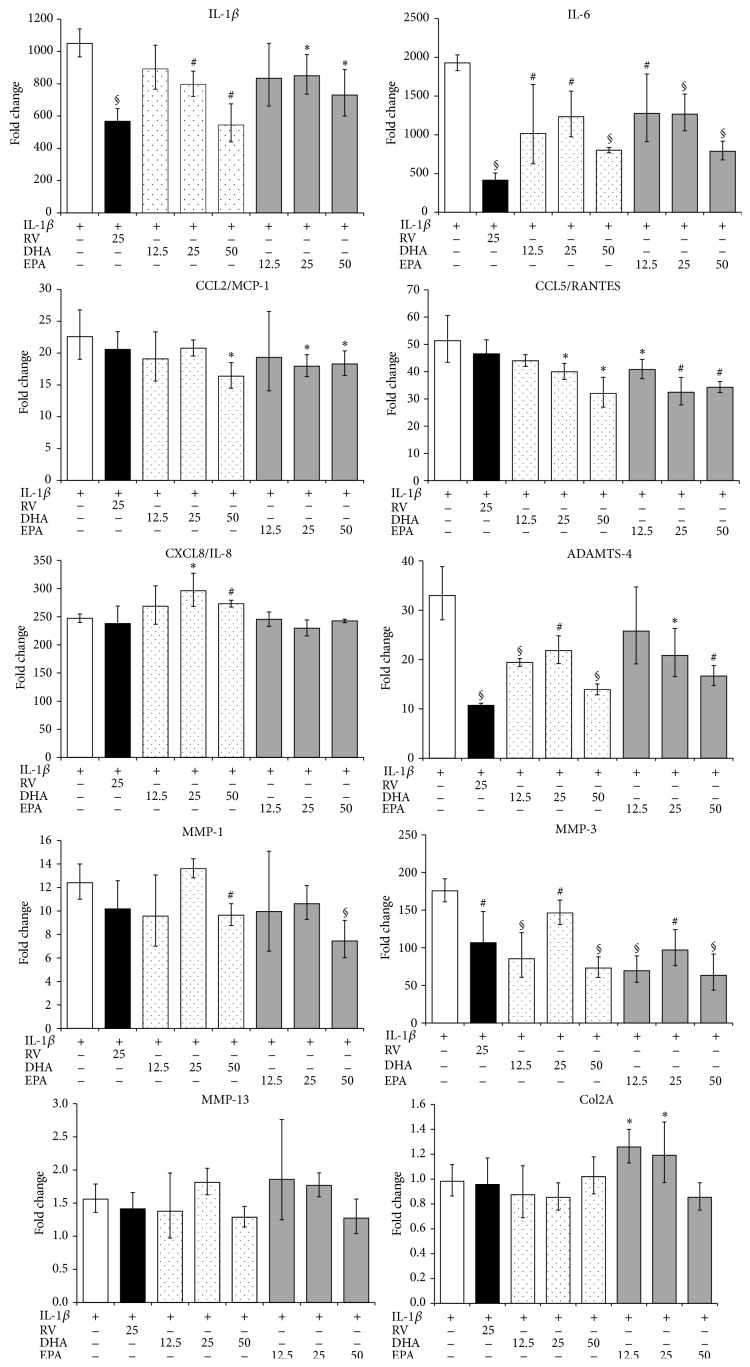
Modulation of gene expression in IL-1*β* activated human chondrocytes. Cells were activated with IL-1*β* with or without the indicated substances for 4 h. Gene expression was quantified by RT-PCR and the data expressed as fold change compared to levels observed in unstimulated cells. ^∗^ *p* < 0.05, ^#^ *p* < 0.01, and ^§^ *p* < 0.001 (IL-1*β* only versus IL-1*β* + substance).

Following activation with IL-1*β*, human chondrocytes (normal human articulocytes from knee, NHAC-kn) expressed various pathophysiological markers of osteoarthritis (OA) and enzymes that degrade the extracellular matrix (ECM) [28–30]. We treated normal human chondrocytes with IL-1*β*, the pathophysiological inducer of OA, and investigated the effect of RV and PUFAs on biological markers of OA. MMPs and ADAMTS, interleukins, and chemokines were upregulated in IL-1*β* treated chondrocytes [20]. In the presence of *ω*-3 PUFAs or resveratrol, gene expression of OA markers was significantly altered (Figure 4). Both substances had no significant impact on gene expression of unstimulated NHAC-kn (not shown). *ω*-3 PUFAs markedly blunted IL-1*β* and IL-6 expression in stimulated NHAC-kn cells. In general, EPA and DHA induced similar alterations of gene expression in IL-1*β* activated chondrocytes. Also, MMP-3 and ADAMTS-4 gene expression was mitigated by EPA or DHA, whereas MMP-1 expression was unaltered. MMP-13 was only slightly upregulated in IL-1*β* treated chondrocytes. Under these conditions, *ω*-3 PUFAs had no significant impact on MMP-13 mRNA levels. Conversely, expression of CCL5/RANTES and CXCL8/IL-8 was drastically upregulated in activated NHAC-kn cells. Concomitant treatment with EPA or DHA led to a mitigated expression of CCL5/RANTES, but they had no effect on CXCL8/IL-8 (Figure 4). EPA positively influenced chondrocyte anabolism, since the expression of Col2A and therefore the synthesis of ECM elements were increased. RV substantially altered the expression of MMP-3 and ADAMTS-4 in IL-1*β*-activated NHAC, whereas its impact on chemokine gene expression was not significant. Therefore, in comparison with *ω*-3 PUFAs, RV is predicted to have a limited impact on cartilage erosion.

## 4. Discussion 

In this study we investigated whether dietary constituents altered the* in vitro* inflammatory response of human leukocytes from healthy individuals. RV was anti-inflammatory, since it reduced, for instance, PGE_2_ and nitric oxide in human PBL [19] and macrophage cell lines. Yet, it had opposite effects on IL-6 produced by human PBL [9, 19, 31, 32]. RV and *ω*-3 PUFAs had similar effects on the production of IL-1*β* and IL-6 but markedly differed in the impact on, for example, CCL2/MCP-1, CCL4/MIP-1*β*, or PGE_2_ production. *ω*-3 PUFAs induced striking changes in eicosanoid metabolites [33], modulated the inflammatory response in human PBLs via PPAR and NF-*κ*B pathways [2, 8, 15, 34–36], and orchestrated the resolution of inflammation [4]. Since EPA and DHA are precursors of resolvins, they might accelerate the resolution of inflammation. The present* in vitro* study is the first report where effects of *ω*-3 PUFAs on a large panel of chemokines were investigated. EPA or DHA markedly increased CXCL8/IL-8 but blunted the secretion of CCL5/RANTES. Plausibly, *ω*-3 PUFA might increase neutrophil recruitment in the early inflammatory phase and attenuate migration of activated T lymphocytes during resolution. Previous studies showed modest effects of *ω*-3 PUFAs on inflammatory markers [37, 38]. A 12-week fish-oil supplementation did not significantly affect plasma cytokine and chemokine concentrations, although an overall trend for an increase of inflammatory markers was observed [39] and gene expression profiles of chemokines and cytokines in peripheral blood mononuclear cells (PBMC) were affected [5, 40]. DHA levels in PBMC were inversely related to IL-1*β* and IL-6 production [13] and diminished IL-1*β* and TNF-*α* secretion by activated PBLs* in vitro* [41]. Incubation of unstimulated PBLs with EPA upregulated the expression of IL-6 and CXCL8/IL-8 [42]. Since in the present study *ω*-3 PUFAs were added shortly before the stimulation of PBLs, they might alter the release of cellular arachidonic acid and the subsequent production of eicosanoid metabolites like PGE_2_ and anti-inflammatory prostaglandins like PGD_2_. These results suggest that acute supplementation with EPA and DHA might transiently disturb homeostasis in PBLs* in vitro* and* ex vivo*, whereas during long-term supplementations cells no longer sense subtle homeostatic changes. Cytokines and chemokines critically determine macrophage differentiation and function [43]. In addition, the T_h_1 and T_h_2 cell development is orchestrated by IL-6, IL-12, TNF-*α*, and chemokines and their receptors [44]. Since immune cells sense, and respond to, the presence of RV and *ω*-3 PUFAs by changes in cytokine and chemokine production, we hypothesize that these substances influence the differentiation of M2 macrophages and T_h_2 cells [45]. For instance, an increase of IL-6 is expected to favour the differentiation of M2 macrophages [46].

In this study we also show that *ω*-3 PUFA modulated mRNA levels in human chondrocytes that were activated with the pathophysiological mediator IL-1*β*. These cells are used as an appropriate* in vitro* model for chronic inflammation, which is a typical feature of osteoarthritis. Notably, expression levels of MMPs, ADAMTS-4, and interleukins were reduced. Similar data were obtained when chondrocytes were incubated with rose hip that also contained significant amounts of free fatty acids including EPA or DHA [47]. Other studies in bovine chondrocytes and cartilage explants have identified that long-term* in vitro* treatment of chondrocytes with conjugated linoleic acids or EPA mitigated the production of PGE_2_ and nitric oxide [48]. In bovine cartilage explants and chondrocytes, EPA affected ECM degradation, since it reduced glycosaminoglycan and collagen II release [49] and reduced gene expression of enzymes involved in OA [50]. Collectively, RV was less active on chondrocytes than *ω*-3 PUFAs.

From these data we infer that RV and *ω*-3 PUFAs modulate acute (in PBLs) and chronic inflammation (in chondrocytes) in different ways: RV mitigates early inflammatory events like the production of PGE_2_. *ω*-3 PUFAs augment the amplitude and kinetics of inflammatory events in acute inflammation and its resolution. Yet, both substances diminish inflammatory processes during chronic inflammation.

## 5. Conclusions

*ω*-3 PUFAs and RV differ in the regulation of acute inflammation in leukocytes, but they have common properties in modulating biochemical events related to chronic inflammation of chondrocytes.

## Figures and Tables

**Table 1:** Secreted proteins of unstimulated and stimulated PBLs obtained from different subjects.

Parameter	Unstimulated cells	LPS-stimulated cells	Range	^(2)^ *R* = (RV^(3)^ + LPS)/LPS	^(2)^ *R* = (EPA^(3)^ + LPS)/LPS
	(pg/mL)	(pg/mL)	(pg/mL)		
IL-1*β*	0 ± 0^(1)^	7889 ± 1250^(1)^	2025–18000	2.62 ± 0.30	2.76 ± 0.94
IL-6	1 ± 1	111644 ± 19519	51300–209000	1.76 ± 0.30	2.29 ± 0.51
IL-10	1 ± 1	1279 ± 230	388–2530	1.29 ± 0.35	1.06 ± 0.21
IL-12 (p70)	0 ± 0	14 ± 7	0–73	—	—
TNF-*α*	0 ± 0	3499 ± 1300	418–12750	0.86 ± 0.15	1.44 ± 0.65
CCL2/MCP-1	1 ± 1	724 ± 163	363–2065	0.77 ± 0.26	0.95 ± 0.14
CCL3/MIP-1*α*	1 ± 1	14718 ± 2698	2720–28250	1.51 ± 0.56	1.27 ± 0.47
CCL4/MIP-1*β*	182 ± 31	68950 ± 12844	33300–162500	1.75 ± 0.46	0.81 ± 0.17
CCL5/RANTES	536 ± 117	6013 ± 1978	670–20300	1.55 ± 1.57	0.77 ± 0.14
CCL11/Eotaxin	28 ± 8	448 ± 82	78–878	1.16 ± 0.11	1.04 ± 0.09
CXCL8/IL-8	311 ± 35	298980 ± 50712	108800–614000	0.79 ± 0.35	2.10 ± 0.60
PGE_2_	121 ± 15	3437 ± 499	1680–6249	0.11 ± 0.17	2.10 ± 0.64

^(1)^Mean ± SEM of values obtained from PBLs of 8 different subjects (each done in duplicate).

^(2)^Ratio: (metabolites produced by PBLs treated with substance + LPS)/metabolites produced by PBLs treated with LPS. Mean ± SD of triplicate values obtained from PBLs of 4 different subjects.

^(3)^25 *μ*mol/L RV, 20 *μ*mol/L EPA.
